# Mapping cis-regulatory elements in the midgestation mouse placenta

**DOI:** 10.1038/s41598-021-01664-x

**Published:** 2021-11-16

**Authors:** Rebekah R. Starks, Haninder Kaur, Geetu Tuteja

**Affiliations:** 1 Genetics, Development, and Cell Biology, Iowa State University, Ames, IA 50011 USA; 2 Bioinformatics and Computational Biology, Iowa State University, Ames, IA 50011 USA

**Keywords:** Epigenomics, Gene regulation

## Abstract

The placenta is a temporary organ that provides the developing fetus with nutrients, oxygen, and protection in utero. Defects in its development, which may be caused by misregulated gene expression, can lead to devastating outcomes for the mother and fetus. In mouse, placental defects during midgestation commonly lead to embryonic lethality. However, the regulatory mechanisms controlling expression of genes during this period have not been thoroughly investigated. Therefore, we generated and analyzed ChIP-seq data for multiple histone modifications known to mark cis-regulatory regions. We annotated active and poised promoters and enhancers, as well as regions generally associated with repressed gene expression. We found that poised promoters were associated with neuronal development genes, while active promoters were largely associated with housekeeping genes. Active and poised enhancers were associated with placental development genes, though only active enhancers were associated with genes that have placenta-specific expression. Motif analysis within active enhancers identified a large network of transcription factors, including those that have not been previously studied in the placenta and are candidates for future studies. The data generated and genomic regions annotated provide researchers with a foundation for future studies, aimed at understanding how specific genes in the midgestation mouse placenta are regulated.

## Introduction

The placenta is an essential organ during development, providing the fetus with adequate nutrients, oxygen, and protection during gestation. Dysfunctions of gene regulation in the placenta can have detrimental effects on the health of the mother and fetus, as well as lead to a variety of placental disorders such as preeclampsia^1^, placenta accreta^2^, or miscarriage^3^. Mouse is a commonly used model to understand the genetic regulation governing the development and function of the human placenta^4^. In the mouse, placental abnormalities are strongly associated with embryonic lethality during midgestation, including lethality at embryonic day (e)9.5^5^, emphasizing the importance of studying gene regulation at this developmental stage. At e9.5, the ectoplacental cone (EPC) is still present, and placental labyrinth formation has initiated with the fusion of the allantois and chorion. The labyrinth space is filled with fetal blood vessels^6,7^ and becomes the site of exchange between the maternal and fetal blood supply. While the placenta is not fully developed at this stage and still contains progenitor cells, understanding what regulates the gene expression patterns at e9.5 will provide a snapshot of the mechanisms that drive mature placenta formation.

Several factors contribute to gene regulation, including transcription factors, cis-regulatory elements, and epigenetic modifications. Post-translational modifications on histones have been found to be important for the regulation of gene expression during development, and in response to environmental stimuli. Perturbations in histone modifications can alter the structure of chromatin, preventing interactions between specific regulating chromatin factors^8^ and have been implicated in development^9^ and cancer^10^.

Histone acetylation is often associated with gene activation. Acetylation of lysine-27 on histone H3 (H3K27ac) is enriched in active gene promoters and in active enhancers^11–13^, distinguishing such regions from poised enhancers^12^. Tri-methylation of the same lysine on the same histone (H3K27me3) has generally been associated with gene repression^11,14^. Mono-methylation and tri-methylation of lysine-4 on histone H3 (H3K4me1/H3K4me3) have instead been associated with specific genomic regions. H3K4me3 is typically enriched at the transcription start site (TSS) of active genes^13^ while H3K4me1 has been found to mark enhancers^12,13,15^.

Though each histone modification individually contributes to our understanding of gene regulation, when combined, these modifications can annotate the genome in more detail. For example, though H3K4me1 marks enhancers, the presence of H3K27ac with H3K4me1 marks active enhancers^12^. H3K4me3 has been found at regions that are also frequently marked with either H3K27ac or H3K27me3. When H3K4me3 and H3k27me3 are identified together, these bivalent regions are generally thought to be associated with poised genes important during development^16^.

Previous studies have used multiple histone modifications to better understand the regulatory landscape of the mouse placenta. Shen et al.^17^ combined RNA-seq data and ChIP-seq data for several histone modifications, RNA polymerase II, and CTCF from e14.5 placenta and 18 other tissues to identify tissue-specific enhancers and promoters. Another study used histone modifications to identify promoters and enhancers in mouse and rat trophoblast stem cells^18^, focusing on identifying differences in the species-specific enhancers to gain insight into placental evolution. Other studies have also used histone modification ChIP-seq in placental cell lines to identify regulatory elements that are bound by transcription factors (TFs) or to identify regulatory elements that may control TF expression^19,20^. However a thorough annotation of the regulatory landscape in the e9.5 mouse placenta, after the chorion and allantois have merged and when the labyrinth and fetal vasculature are developing to allow adequate exchange of material^21^, has not been done.

Therefore, to identify cis-regulatory elements involved in gene regulation in the e9.5 placenta, we performed ChIP-seq to identify regions marked with the H3K27me3, H3K4me1, or H3K4me3 histone modifications. Using this data and a published H3K27ac ChIP-seq dataset^22^, we employed ChromHMM^23^ to annotate the e9.5 placental genome, identifying potential repressors, enhancers, active promoters, and bivalent regions. Overall, these data provide a valuable resource for researchers trying to understand gene regulation in the midgestation placenta.

## Results

### ChromHMM defines chromatin states in e9.5 mouse placenta

To analyze the interplay of certain histone modifications and to understand regulatory mechanisms in the murine midgestation placenta we generated ChIP-seq data for three histone marks in placenta at e9.5. We focused on histone modifications known to be associated with genomic regions with different regulatory functions: H3K27me3 (facultative heterochromatin ^8,13,14^), H3K4me1 (enhancers^8,13,15^), and H3K4me3 (promoters^8,13,15^). After aligning, filtering, and calling peaks on each sample, we used principal component analysis (PCA) on peak scores and saw that replicates cluster based on the histone modification that was assayed and data from each histone modification were well separated (Supplementary Figure S1). We also incorporated previously published H3K27ac^22^ ChIP-seq data, which marks active enhancers^12,13^ and promoters^11,13^.

**Figure 1:**
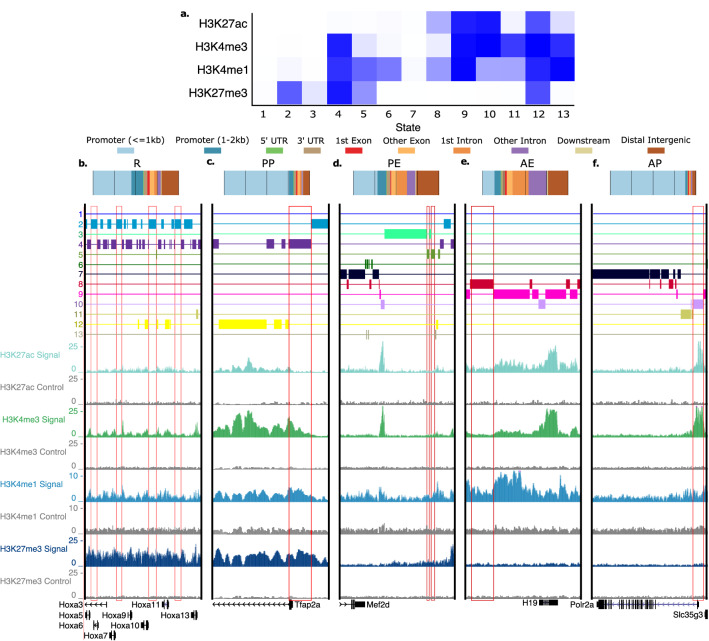
ChromHMM defined chromatin states in e9.5 mouse placenta. (**a**) ChromHMM heatmap displaying the amount of activity of each histone modification in the identified states. (**b**–**f**) Top: Genomic feature locations of regions within each identified state. Bottom: UCSC Genome Browser view showing example(s) of genomic region(s) (red box(es)) associated with each state. (**b**) State 2—repressed (R) (**c**) State 4—poised promoter (PP) (**d**) State 5—poised enhancer (PE) (**e**) State 8—active enhancer (AE) (**f**) State 10—active promoter (AP). Control signal tracks are for input DNA.

We used ChromHMM^23^ to associate each region of the genome with one of 13 states, based on the presence of aligned reads for each histone modification in the region (Fig. 1a). ChromHMM discovers combinatorial patterns of histone marks throughout the genome by applying a multivariate hidden markov model, based on the presence or absence of each histone mark. Regions containing the same pattern of histone marks are placed in the same state. To map the ChromHMM state to an actual chromatin state with a cis-acting function (e.g. promoter, repressor, enhancer), we looked more closely at the histone modifications present in a state, as well as the location of the regions within the state. We focused on states enriched for patterns of histone modifications that are well characterized (States 2, 4, 5, 8, 10). To make our analysis more stringent, we ensured that each region marked as belonging to a state also contained significant peaks for the histone modifications associated with the state (see “Methods”).

State 2 regions contained high H3K27me3 activity. Regions in this state were distributed across the genome (Fig. 1b(top)), consistent with a previous study that found a similar pattern for the H3K27me3 mark^24^. State 2 is therefore likely associated with gene repression (R). An example of a genomic region in the R state is shown around the *Hoxa* cluster of genes, which are globally silenced during development^25^ (Fig. 1b(bottom)). Though the *Hoxa* cluster region generally shows H3K27me3 activity, we also observe narrow state 4 regions at the transcription start site (TSS) of certain *Hoxa* genes, such as *Hoxa9, Hoxa10,* and *Hoxa13,* which have known roles in placenta development^26–28^*.* State 4 regions contain high levels of H3K27me3, H3K4me1, and H3K4me3 activity and are most commonly found in promoter regions (Fig. 1c(top)), likely representing poised, or bivalent, promoters (PP) which have previously been found to be marked by these modifications^29^. An example of a genomic region in the PP state is shown at the TSS of the *Tfap2a* gene (Fig. 1c(bottom)), which plays a role in trophoblast differentiation^30^ and has higher expression within the placenta prior to e9.5^22^ (Supplementary Figure S2). State 5, on the other hand, has H3K27me3 and H3K4me1 activity, as well as very low H3K4me3 activity. Regions in this state are also found at promoters, but more frequently at intergenic and intronic regions (Fig. 1d(top)), likely representing poised enhancers (PE). An example of a genomic region marked by the PE state is shown downstream of the *Mef2d* gene (Fig. 1d(bottom)), which plays a role in human trophoblast invasion^31^. State 8 regions have both H3K27ac and H3K4me1 activity, a pattern which has previously been found at active enhancers^12^. The majority of this state is found at intergenic and intronic regions (Fig. 1e(top)), further supporting that state 8 marks active enhancers (AE). We observe that the *H19* enhancer is in the AE state^32^ (Fig. 1e(bottom)). *H19* is an imprinted gene which may play a role in trophoblast proliferation and invasion^33^. Finally, state 10 regions contain high H3K27ac and H3K4me3 signals, marks previously shown to indicate active promoters when identified together^13,29,34^. Indeed, we observed that regions within this state are most commonly found in promoter regions (Fig. 1f(top)), and we therefore refer to state 10 as the active promoter (AP) state. A region in the AP state is observed at the TSS of the housekeeping gene, *Polr2a*^35^(Fig. 1f(bottom))*.*

We created a publicly available session (e9.5Placenta_HistoneModification) in the UCSC genome browser containing averaged ChIP-seq read tracks for each histone modification as well as ChromHMM state information.

### Poised and active promoter states are associated with distinct gene sets

**Figure 2:**
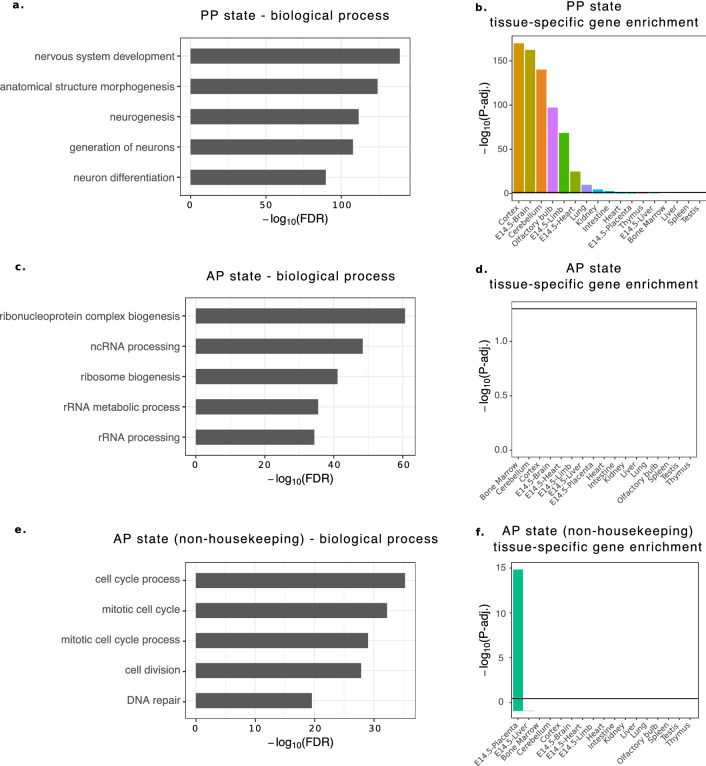
Poised promoters are associated with neuronal genes while active promoters tend to mark housekeeping genes. (**a**) Genes associated with poised promoters are enriched for biological processes related to neuron development. (**b**) Genes associated with the PP state are enriched for genes with brain-specific expression (cortex, e14.5 brain, cerebellum, and olfactory bulb). (**c**) Genes associated with active promoters are enriched for biological processes related to general cellular functionality. (**d**) Genes associated with the AP state do not have tissue-specific gene expression, potentially indicating they are house-keeping genes. (**e**) Non-housekeeping AP state genes are enriched for genes involved in the cell cycle process. (**f**) Non-housekeeping AP state are enriched for genes with placenta-specific expression.

First, we investigated genes associated with the PP state using gene ontology analysis and found that nervous system and neuronal development terms were enriched (Fig. 2a). Many of these genes also have brain-specific expression (Fig. 2b). In studies performed in embryonic stem cells, poised or bivalent promoters were found to mark transcription factors important for development^36–39^. Though we found several neuronal genes in the PP State, we also found a subset of genes that code for TFs with roles prior to mid-gestation, including stem cell differentiation (SATB2^40^), trophoblast differentiation (HAND1^41^, ASCL2^42^, TCFAP2A^43^), and trophoblast migration (FOSL1^44^). According to previously published RNA-seq data from the EPC at both e7.5 and e9.5 (which also includes the chorion)^22^ and the placental disc at e12.5^45^, each of these genes shows decreasing expression as placental development progresses, suggesting these bivalent regions are becoming stably repressed (Supplementary Figure S2).

Next, we investigated genes associated with the AP state, and found enrichment for general cell functionality terms (Fig. 2c) and no enrichment for genes with tissue-specific expression (Fig. 2d), potentially indicating the presence of a high number of house-keeping genes. Previous studies have observed that genes with housekeeping functions have narrow H3K4me3 peaks at the TSS^46^ or highly accessible regions within their promoter^47,48^. Therefore, using previously published ATAC-seq^47^ data generated in the e9.5 placenta, we intersected accessible peaks with each of the five analyzed states. We found that the AP state has a much higher percentage of regions overlapping accessible regions than all the other states, further indicating a house-keeping status (Supplementary Figure S2). We then overlapped a list of housekeeping genes with the AP state and determined that 80% of the housekeeping genes are found within this state, further confirming that the strong H3K27ac and H3K4me3 signal of the AP state mark housekeeping genes. However, housekeeping genes only account for 46% of the AP genes. To determine the role of non-housekeeping AP genes, we removed the housekeeping genes and repeated the ontology and tissue-specific gene analysis. When looking at all of the AP non-housekeeping genes we see that the group is associated with the cell cycle process (Fig. 2e) and that this group is enriched for genes with placenta-specific expression (Fig. 2f). We found several interesting genes within this group, such as *Gjb3*, *Peg3*, and *Dusp9*, which are known to be important for mouse placental development. *Gjb3* knockout mice have reduced labyrinth and spongiotrophoblast size at e9.5, and *Dusp9* knockout mice do not form a proper labyrinth layer^49,50^. *Peg3* knockout mice have defects in the spongiotrophoblast layer and in the glycogen cell lineage in male fetuses^51^.

Finally, as expected, when comparing expression between AP and PP state genes, we see that genes with poised promoters have significantly lower expression than genes with active promoters (p-value < 2.2e−16) (Supplementary Figure S2).

### Investigation of the repressed state and the enhancer state

**Figure 3:**
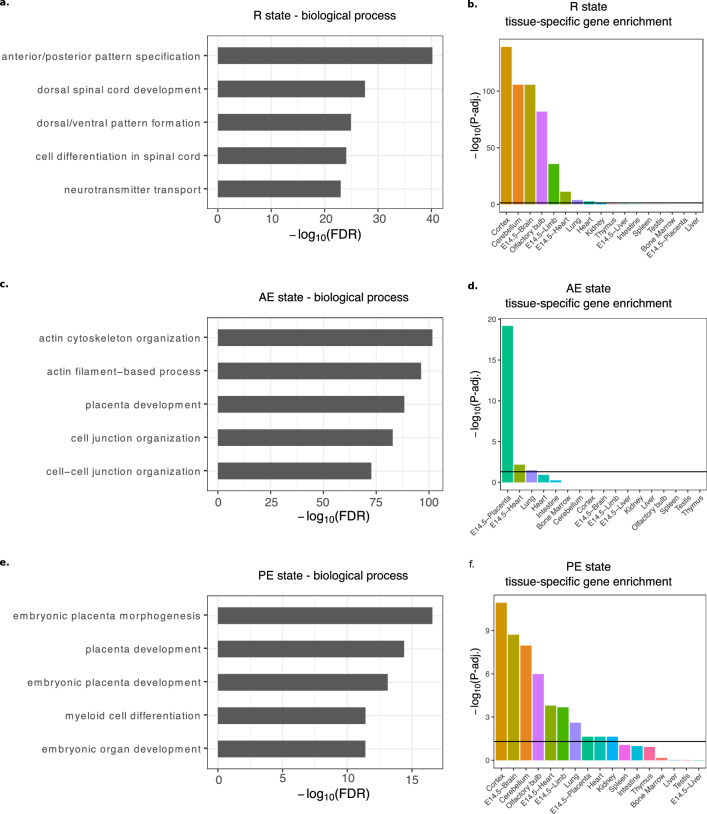
Repressed regions are associated with neuronal terms while enhancers are associated with placental development. (**a**) Genes associated with repressed regions are enriched for biological processes related to spinal cord development and pattern formation. (**b**) Genes associated with the R state are enriched for genes with brain-specific expression (cortex, e14.5 brain, cerebellum, and olfactory bulb). (**c**) Genes associated with active enhancers are enriched for biological processes related to placental development and cytoskeleton organization. (**d**) Genes associated with the AE state are strongly enriched for genes with placenta-specific expression. (**e**) Genes associated with poised enhancers are enriched for biological processes related to placental development. (**f**) Genes associated with the PE state are enriched for genes with tissue-specific expression in multiple tissues.

Next, we investigated the functions and expression levels of genes associated with the non-promoter states, i.e. potential repressive and enhancer regions. We found that genes associated with regions in the R state were enriched for terms related to pattern specification, spinal cord development, and neurotransmitter transport (Fig. 3a). This set was also enriched for genes with brain-specific expression (Fig. 3b), similar to the PP state. These results agree with a previous study that associated H3K27me3 activity with neuronal genes in the e9.5 placenta^47^. Genes associated with regions in the AE and PE states are associated with placental development (Fig. 3c,e), however, those associated with active enhancers are enriched for actin cytoskeleton and cell junction organization (Fig. 3c) as well, processes important for trophoblast cell differentiation and cellular adhesion^52,53^. When using the Mouse Phenotype Single KO ontology, we observe enrichment of several terms related to placenta morphology (abnormal placenta morphology, abnormal trophoblast layer morphology, and abnormal placenta labyrinth morphology) and embryonic lethality (embryonic lethality prior to organogenesis) associated with the AE state genes, whereas such placenta terms were not enriched for PE state genes (Supplementary Figure S3). AE genes, such as *Cdkn1c*, *Il11ra1*, *Rbpj*, *Birc6*, and *Ncoa6* all play important roles in placental development and morphology, which has been shown using mouse models^54–58^. Tissue enrichment analysis further showed that AE state genes have placenta-specific gene expression (Fig. 3d), whereas the PE set contained genes that are expressed in multiple tissues (Fig. 3f). This is expected since active enhancers are known to drive tissue-specific gene expression^59^.

When we compare the expression level of genes associated with non-promoter states, we see that genes with an associated AE state have the highest expression, significantly higher than those genes with an associated PE state (p-value < 2.2e-16) and, unsurprisingly higher than genes with an associated R state (p-value < 2.2e-16) (Supplementary Figure S3).

### Identification of transcription factors regulating AE regions

**Figure 4:**
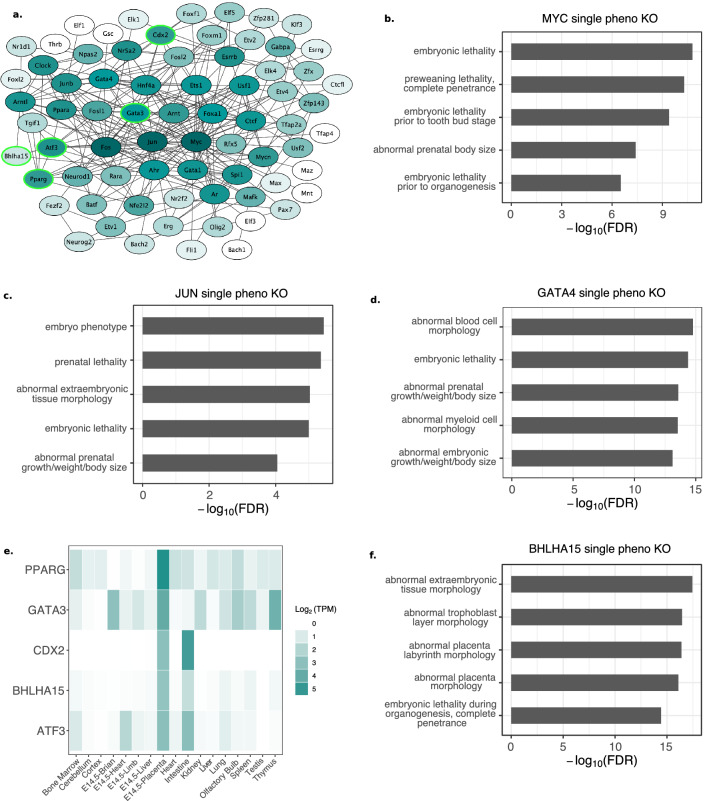
Transcription factors enriched in active enhancers. (**a**) Protein–protein interaction network comprised of TFs enriched within AE state intergenic or intronic regions. The darker the color, the higher the number of connections (edges). Light green outline indicates a gene with placenta-specific expression as defined by TissueEnrich. (**b**) The MYC TF targets genes are associated with embryonic lethality and prenatal size. (**c**) The JUN TF targets genes are associated with embryonic lethality and size. (**d**) The GATA4 TF targets genes associated with embryonic size, embryonic lethality, and cell morphology. (**e**) TissueEnrich heatmap showing the genes that have placenta-specific gene expression patterns. (**f**) The BHLHA15 TF targets genes associated with placental morphology.

Because enhancers generally drive tissue-specific gene expression^59^, we used HOMER^60^ to search for known TF motifs enriched in intergenic or intronic AE regions. We only analyzed TFs with a q-value ≤ 0.01, that are also recognized by STRINGdb^61^, leading to 76 unique TF motifs that were enriched within the AE regions (Supplementary Table S1). Of these, 72 were connected in a protein–protein interaction network generated using STRINGdb (Fig. 4a). The hub TFs (see “Methods”) in this network were MYC, FOS, JUN, and GATA4, most of which play important roles in placental development^44,62,63^. We see a large overlap between FOS and JUN predicted target regions, explained by the high similarity in motifs, with almost all JUN predicted binding sites in the same region as a FOS predicted binding site (Supplementary Figure S4). Therefore, we focus on the MYC, GATA4, and JUN target regions since JUN has a stronger motif than FOS. The genes targeted by all of these TFs are related to embryonic lethality and abnormal embryonic size, a common effect of abnormal placental development or function^64,65^ (Fig. 4b–d).

We identified several TFs with placenta-specific gene expression in the network including PPARG, GATA3, CDX2, ATF3, and BHLHA15 (Fig. 4a -green outlined; Fig. 4e). PPARG^66^ and GATA3^67,68^ are known to be involved in promoting trophoblast differentiation, while CDX2 is required for trophoblast stem cell maintenance^68,69^. ATF3 has been found to be decreased in placentas from preeclamptic pregnancies in response to hypoxia^70^. We predict that one of these expressed placenta-specific transcription factors, BHLHA15, may have a role in placenta that has not been fully elucidated. When we used the 1,182 predicted binding sites of BHLHA15 to analyze the enriched terms associated with its potential target genes, we found that BHLHA15 is associated with 1,733 genes. These genes, such as *Cdkn1c*, *Dusp9*, *Dlx3*, and *Pdgfb* (Supplementary Table S2), are associated with terms related to abnormal placental and labyrinth morphology (Fig. 4f) and are either known to be important for proper placental development, or have been associated with pregnancy disorders^50,54,71,72^.

## Discussion

Using ChIP-seq for multiple histone modifications we annotated chromatin states in the e9.5 mouse placental genome, identifying putative repressors, enhancers, and promoters. We also identified a network of TFs enriched within active enhancers, that were predicted to target placenta-specific genes, some of which have not been well-studied in placental development. While other studies have used histone modifications to identify the activity or location of regulatory elements in the e14.5 placenta^17^ or trophoblast stem cells^18–20,73^, our study is the most thorough annotation of the e9.5 placenta. The data we generated is useful for the research community and could be adapted to many other analyses. Our pipeline, which includes ChromHMM, could be compared with data previously generated in trophoblast stem cells, trophoblast giant cells, and e14.5 placenta, to determine how the chromatin landscape changes during placental development. The data we generated could also be used to identify genomic elements regulating genes of interest in the placenta, or be combined with other datasets, such as TF ChIP-seq, to determine whether the TF is associated with active promoters or correlated with a specific histone mark. Additional datasets, such as those generated from placentas exposed to environmental stressors or affected by genetic mutation could also be combined with our data to help identify regions sensitive to these affects.

We recognize certain limitations associated with this study. First, though general characteristics of histone modifications have been established, these characteristics do not always hold true. For example, the repressive H3K27me3 mark has been found within promoters of highly expressed genes^74^, and H3K4me3 is generally associated with active transcription, but not all genes marked are transcribed^75^. This is evident when we look at the expression of the AP and PP states. Though AP state genes are significantly more highly expressed, there is a wide range in expression level and many genes of the PP state are also considered expressed.

A second limitation we observed is that some states seem to be residual noise from their neighboring, stronger states. For example, state 11 shows similar marks to the AP state, without the H3K27ac mark. Approximately 50% of state 11 regions are within 500 bp of an AP state region, potentially indicating they are flanking the regions marked by a narrow H3K27ac peak. To further classify and distinguish states to characterize the genome, it would be beneficial to profile several other histone modifications such as H3K36me3, which marks exons of expressed genes^76^, or H3K9me3, marking constitutive heterochromatin^13^. Other datasets can also help annotate cis-regulatory regions—such as RNA PolII ChIP-seq, to mark sites of active transcription.

A third limitation is that the previously published data included for placental RNA-seq were not from mice of the same genetic background. The e7.5 EPC and e9.5 placenta (EPC and chorion) RNA-seq data came from CD-1 mice, but the e12.5 placental disc RNA-seq data was from a cross of C57BL6 females and CAST/Ei males. Genetic background can impact placental morphology^77^, and it would therefore be more robust to obtain RNA-seq data from several developmental timepoints that have been collected from the same strain using the same methodologies.

We found that some states contained what might be considered as conflicting histone marks. For example, ChromHMM identified a state (state 12) characterized by the presence of all four histone modifications (Fig. 1a). Since some of these marks are not as frequently localized together (H3K4me3/H3K4me1 and H3K27ac/H3K27me3), the biological relevance of this finding remains to be understood. One possibility is that many of these regions play cell-specific roles within the placenta, and that the region is active in certain cell types and repressed in other cell types. Different trophoblast cells likely have distinct regulatory profiles, which could be resolved using single-cell ChIP-seq. As described above, though PP genes are generally thought to have low expression, we identified a large range of expression within this set of genes. This is possibly due to the dual nature of the PP genes: while some are becoming active, others are becoming more repressed as development progresses. Several studies have found that bivalent genes in stem cells resolve to be either stably expressed or repressed upon differentiation, though some retain their bivalent nature^36,38,39^. We identified several PP genes involved in differentiation that showed decreasing expression as placental development continued, including *Satb2*, *H*and1, *Ascl2*, and *Tcfap2a* (Supplementary Figure S2a).

Neurogenesis related terms were also enriched when analyzing PP genes. Further study of these genes would aid in understanding why they annotated with a poised status in the placenta. It is possible that the poised status may be necessary to keep such genes turned off in non-neuronal tissues such as the placenta, or that the genes are marked as poised because they were previously in a pluripotent state^78^. Another possibility is that these genes may have similar roles in the development of the placenta and neurons, such as Dll4 and Notch1, key members of the Dll4/Notch signaling pathway which play a role in angiogenesis in the retina^79^, the placenta^80^, and in neurogenesis^81,82^. Interestingly, fetal sex has been shown to impact neurolation^83^, as well as placental development and gene expression, both in normal development and following in utero environment changes^84^. For example, sex-specific changes in placental gene regulation has been seen in response to maternal diets^85–87^ and stress hormones^88,89^. Therefore, it is possible that the regulatory landscape of the placenta differs when it is obtained from male versus female embryos, which is not addressed in our study. Because we pooled placentas across multiple biological replicates and see that data is separated by histone modification (Supplementary Figure 1), we do not expect that fetal sex influenced the enriched biological processes we observed. However, by incorporating the fetal sex in future studies, we will be able to better understand gene regulation during placental development and the impact of sex-specific regulation.

In contrast to the PP genes, AP genes were associated with both housekeeping genes and placenta specific genes such as prolactin genes (*Prl2c3*, *Prl2c5*), and placenta specific protein 1 (*Plac1*). Interestingly, around e12.5 to e14.5 the placenta goes through drastic changes in gene expression. Genes upregulated before this switch, during the developing phase (~ e8.5– ~ e13.5), were found to be enriched for terms related to RNA processing and metabolism as well as those involved in the cell cycle, which are similar to a category of terms associated with the active promoters identified in this study (Fig. 2c,e)^90^. After e13.5, genes involved in cell growth and pregnancy become enriched. These genes were found to be more recently evolved and more species-specific^90^. Since we identify both housekeeping and placenta-specific genes in the AP group, it would be interesting to analyze the cis-regulatory elements from this study for their selection potential^91^, which would further contribute to our understanding of how the cis-elements contribute to placenta evolution^18^, and would provide greater insight into the importance of cis-element associated genes^92^.

Our analysis also led to the identification of TFs enriched within active enhancers that have not previously been studied in the placenta, including BHLHA15 and GATA4. Gene ontology analyses revealed a potentially novel role in labyrinth development for BHLHA15. GATA4 has not been thoroughly investigated in placenta, though GATA3, a protein with a very similar binding site has. GATA3 has been found to be important in trophoblast differentiation^68^, as well as migration and invasion of trophoblast cells^93^. To validate that GATA4 is regulating active enhancers, rather than GATA3, or that BHLHA15 is regulating these enhancers, experimental follow-up is necessary. ChIP-seq could be used to verify their binding regions and gene knockout studies would be important to clarify a role in placental development. Future work should also include luciferase assays to assess how predicted repressors or activators impact reporter gene expression. Finally, chromatin capture experiments could be used to identify the true targets of enhancers, allowing more accurate assessment of the functions being affected by each regulatory region.

In summary, we have created a resource to aid researchers in understanding normal placental development, which is a poorly understood process. Our study highlights regions that, when perturbed, could lead to abnormal placental development due to gene misregulation. Data generated in this study will be useful for the advancement of research in placental development, aiding in the identification of regulatory regions important for proper function of this understudied organ.

## Methods

### ChIP-seq library preparation and sequencing

Fetal placental tissue were dissected from e9.5 timed-pregnant CD-1 mice (Charles Rivers Labs) as described previously^22^. All animal experiments were approved by the Iowa State University Institutional Animal Care and Use Committee (Protocol IACUC-18-350) and conformed to the NIH Guidelines for the Care and Use of Laboratory Animals. To isolate the placenta, implantation sites were extracted from the uterine lining and the ectoplacental cone (EPC) and chorion, which has merged with the allantois to begin forming the labyrinth, were separated from the decidua, yolk sac, umbilical cord, and embryo, then collected. Three placentas were collected from the same litter and pooled into one biological replicate as previously described^47^. However, each biological replicate was from a different litter. ChIP was then performed as previously described for H3K27me3^47^ with 10ug of chromatin and 4ug of H3K27me3 antibody (Millipore 17–622, lot:3070997) for a total of 4 biological replicates (where each replicate is from a different litter). H3K4me1 and H3K4me3 ChIPs were performed as previously described^94^ with the following modifications: 2.5ug of chromatin was precleared with 80ul of Protein G-agarose (Thermofisher 20422) in 1 ml of ChIP dilution buffer and incubated overnight with 2ug of H3K4me1 antibody (Abcam ab8895, lot: GR3264996-2) or 2ug of H3K4me3 antibody (Abcam ab8580, lot: GR3264593-1), generating 6 biological replicates for each antibody. Input DNA (control) was simultaneously prepared from chromatin for each replicate. The enrichment level (fold-change) for each histone modification was quantified using Real-Time qPCR with input DNA for normalization. Enrichment was calculated using the ΔΔCt method with 28S as a reference primer and input DNA as a control. *Syt1* (H3K27me3), *B4galt5* (H3K4me1), and *Ldha* (H3K4me3) (Supplementary Table S3; Supplementary Figure S5) were used as positive controls. Efficiency of primers was calculated by testing four-fold serial dilutions of input DNA (Supplementary Table S3).

ChIP-seq libraries were generated as previously described^95^ with the following modifications: 40ul (80%) of the purified ChIP DNA was used for library preparation for each biological replicate and gel size selection was performed by excising DNA fragments between 250 and 350 bp. 5 ng of input sample libraries were also prepared simultaneously as controls. Libraries were run on the Agilent 2100 bioanalyzer using the high sensitivity DNA kit to ensure proper size selection. Both ChIP and input libraries were diluted to 4 nM and pooled together. Further dilution of the samples and sequencing on Illumina HiSeq 3000 was performed by the Iowa State University DNA facility.

### ChIP-seq data processing

ChIP-seq data for the H3K27ac associated histone mark was obtained from GEO (GSE65807^22^). Read quality for all ChIP-seq samples was determined using FASTQC^96^ (v0.11.7) and reads were aligned to the mm10 genome using Bowtie2^97^ (v2.3.4.1; –very-sensitive). All samples had high average overall alignment rates (Supplementary Table S4). After reads were aligned, they were filtered to remove reads with low mapping quality (≤ 30 MapQ), reads that align to the mitochondrial genome, and duplicates (Picard v2.5.0; http://broadinstitute.github.io/picard). BAM files for each experiment were then merged and converted to bigwig using bamCoverage from deepTools (v2.5.2) for browser visualization^98^.

### ChIP-seq data analysis

Filtered Bowtie2 BAM files were used in ChromHMM^23^ (v1.20) with 13 states, using ChIP-seq data and corresponding input DNA data as controls. The BAM files were also used for peak calling with MACS2^99^ (v2.1.1), either broad peaks (H3K27me3; –broad-g mm-f BAM-q 0.05-bdg-keep-dup all) or narrow peaks (H3K27ac, H3K4me3, H3K4me1; -g mm-f BAM-q 0.05-bdg-keep-dup all). Peaks within 500 bp were merged together using BEDTools2^100^ (v2.27.1; merge) and PCA was run on H3K4me3, H3K4me1, and H3K27me3 biological replicates in order to determine correlation between replicates (Supplementary Figure S1) using the R package ChIPQC^101^ (v1.28.0). Peaks were retained if they occurred in at least half of the corresponding biological replicates and did not overlap problematic regions that have high signal across all ChIP-seq data, likely due to amplification noise^102^. After ChromHMM regions were assigned to states, they were further intersected with MACS2 peaks, and only regions containing peaks for the appropriate histone marks were kept for further analysis. For the repressed (R) state, ChromHMM regions containing an H3K27me3 peak were retained. For the active enhancer (AE) and poised enhancer (PE) states, an H3K4me1 peak had to be present within the ChromHMM region. For the active promoter (AP) and poised promoter (PP) states, an H3K4me3 peak had to be present within the ChromHMM region. The genomic feature that ChromHMM regions were found in, and their location in relation to TSSs were identified using the R package ChIPseeker^103^ (v1.24.0).

### ATAC-seq data processing

ATAC-seq data from the e9.5 placenta (EPC and chorion from CD-1 mice) were obtained from GEO (GSE120599) and processed as previously described^47^. After removing duplicates, MACS2 (-f BAMPE-g mm-keep-dup all-q 0.05) was used to call narrow peaks. Peaks overlapping problematic regions were removed and peaks within 100 bp of each other were merged. Only peaks that were in at least two of the three replicates were kept for analysis.

### RNA-seq data processing

RNA-seq data used in this manuscript were previously published and obtained from the EPC at e7.5, EPC and chorion at e9.5, and the placental disc at e12.5. RNA-seq data was obtained from GEO (GSE65808^22^ (e7.5 and e9.5), GSE124215^45^ (e12.5)) and processed as previously described^47^. Reads were aligned to the mm10 mouse genome using HISAT2^104^ (v2.2.0; default parameters) and transcript abundance was calculated by htseq-count from the HTseq^105^ software package. We then calculated transcript per million (TPM)^106^ for each gene, and TPMs for each gene were averaged across biological replicates.

### Gene ontology and gene associations

Gene ontology enrichment analysis and gene associations with state regions were performed using the Genomic Regions Enrichment of Annotations Tool (GREAT v4.0.4)^107^. The Biological Process ontology and the MGI Single Phenotype KO ontology were used for analysis. For regions associated with promoters, we used the single nearest gene option, associating each region with its nearest gene. Terms were considered significantly enriched if they had a ‘Hyper FDR Q-Val’ ≤ 0.05, a ‘Hyper Fold Enrichment’ ≥ 2 and were associated with at least 5 genes. For distal regions, default GREAT options were used for gene association and enriched terms had to have a ‘Binom FDR Q-Val’ ≤ 0.05 a ‘Binom Fold Enrichment’ ≥ 2 and had to be associated with at least 5 genes. For all figures, the most significant terms based on FDR are shown.

### Transcription factor and network analysis

Enrichment of transcription factors within active enhancer regions was determined using HOMER^60^ (v4.11.1; findMotifsGenome.pl-genome mm10) motif finding software and the mm10 genome. Only significant (q-value ≤ 0.01) transcription factors found were kept for analysis. Mouse transcription factors were identified using the Animal Transcription Factor Database^108^ (v3.0). Targets of enriched transcription factors were also identified using the HOMER software (findMotifsGenome.pl-find).

The STRING database^61^ (v11.0) was used to build protein–protein interaction networks from enriched TFs. Networks were built with a confidence threshold of 0.40 and using all forms of evidence. Networks were analyzed using Cytoscape^109^ (v3.8.0) and hub nodes were defined as the top three nodes with the highest degree (MYC, JUN, FOS) and the top three with the highest betweenness score (MYC, JUN, GATA4).

### Tissue-specific gene enrichment

TissueEnrich^110^ was used to carry out tissue-specific gene enrichment analysis using all of the tissue-specific genes from the mouse ENCODE data as background, and using default parameters. Tissue-specific gene sets with an adjusted p-value ≤ 0.01 (horizontal line on tissue-specific bar plots) were considered enriched, using the hypergeometric test.

## Supplementary Information

Supplementary Information 1.

Supplementary Information 2.

## Data Availability

All data generated in this study have been deposited in the Gene Expression Omnibus under the accession ID GSE179695.
