# Supplementary material for: Mapping cis-regulatory elements in the midgestation mouse placenta
Source: Sci Rep. 2021 Nov 16;11:22331. doi: 10.1038/s41598-021-01664-x (PMC8595355; doi:10.1038/s41598-021-01664-x)
Supplement: Supplementary file 1 — Supplementary Information 1. [file 41598_2021_1664_MOESM1_ESM.pdf]

Mapping cis-regulatory elements in the midgestation mouse placenta

Authors: Rebekah R. Starks[1,2], Haninder Kaur[1], Geetu Tuteja[1,2]*

[1] Genetics, Development, and Cell Biology, Iowa State University, Ames, IA, 50011, USA
[2] Bioinformatics and Computational Biology, Iowa State University, Ames, IA, 50011, USA

*Correspondence: geetu@iastate.edu

SUPPLEMENTARY FIGURES S1-S5

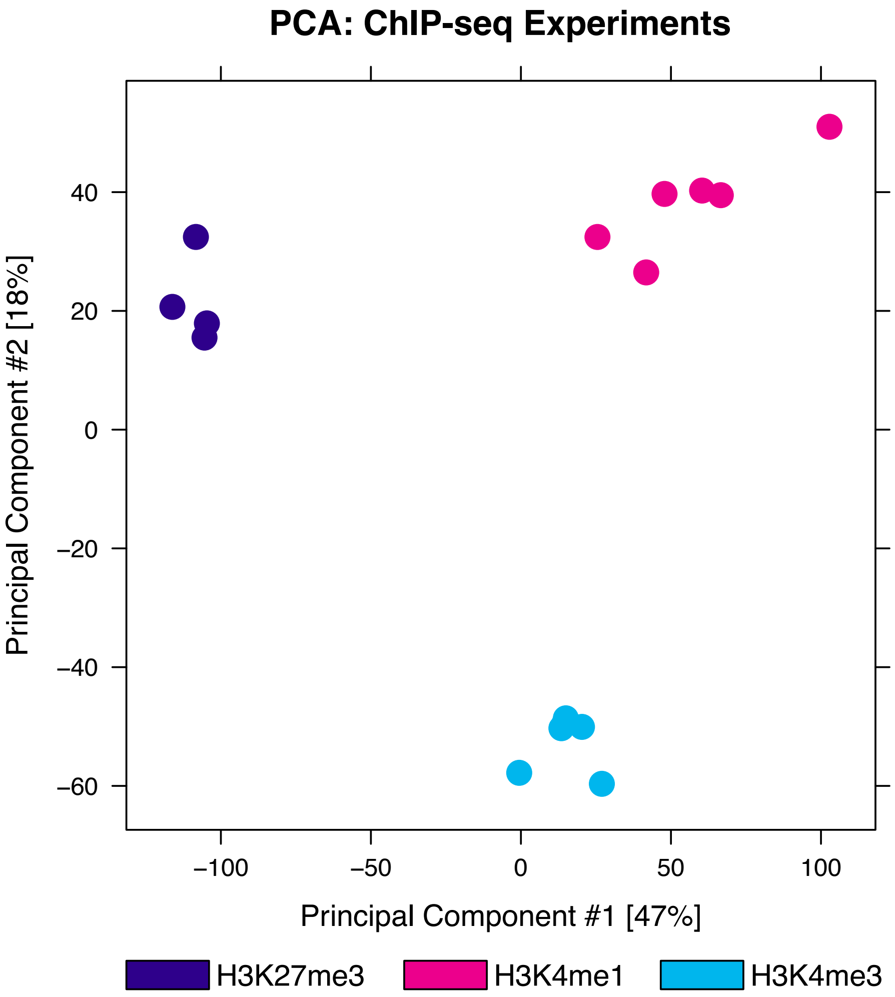

Supplementary Figure S1. PCA plot based on peak scores from H3K4me3, H3K4me1, and H3K27me3 ChIP data. Biological replicates cluster by histone modification that was assayed.

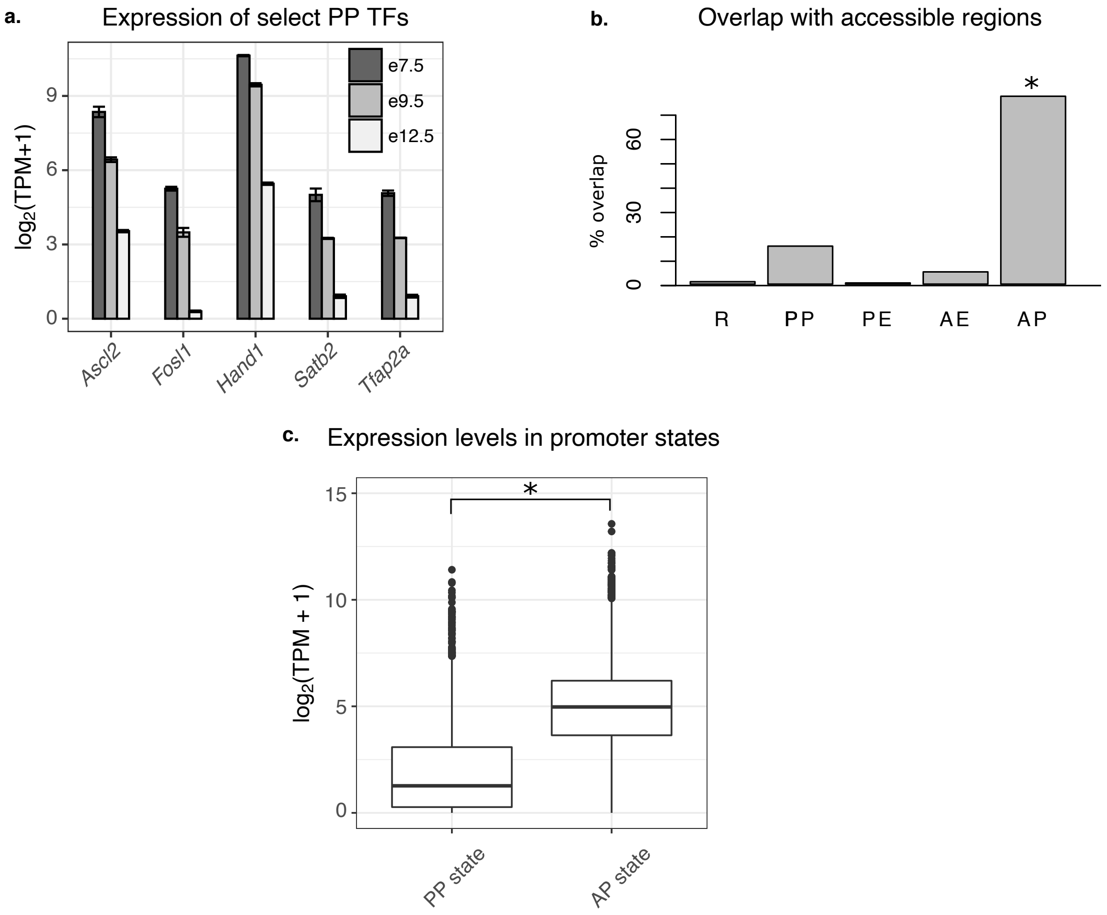

Supplementary Figure S2. (a) Barchart showing the decreasing expression of five PP TFs from e7.5 EPC to e9.5 placenta (EPC + chorion) to e12.5 placenta disc, using previously published RNA-seq data[1,2]. (b) Barchart showing overlap between regions in each state and accessible regions within the e9.5 placenta (EPC + chorion) identified by ATAC-seq[3]. AP regions overlap more than all other states. * represents groups with significant overlap with accessible regions (p-value <0.05; hypergeometric test). (c) Boxplot showing that expression of genes associated with the AP state are significantly higher than expression of genes associated with the PP state using the same e9.5 placenta RNA-seq data used in (a)[1] (* p-value < 0.05; students one-tailed t-test).

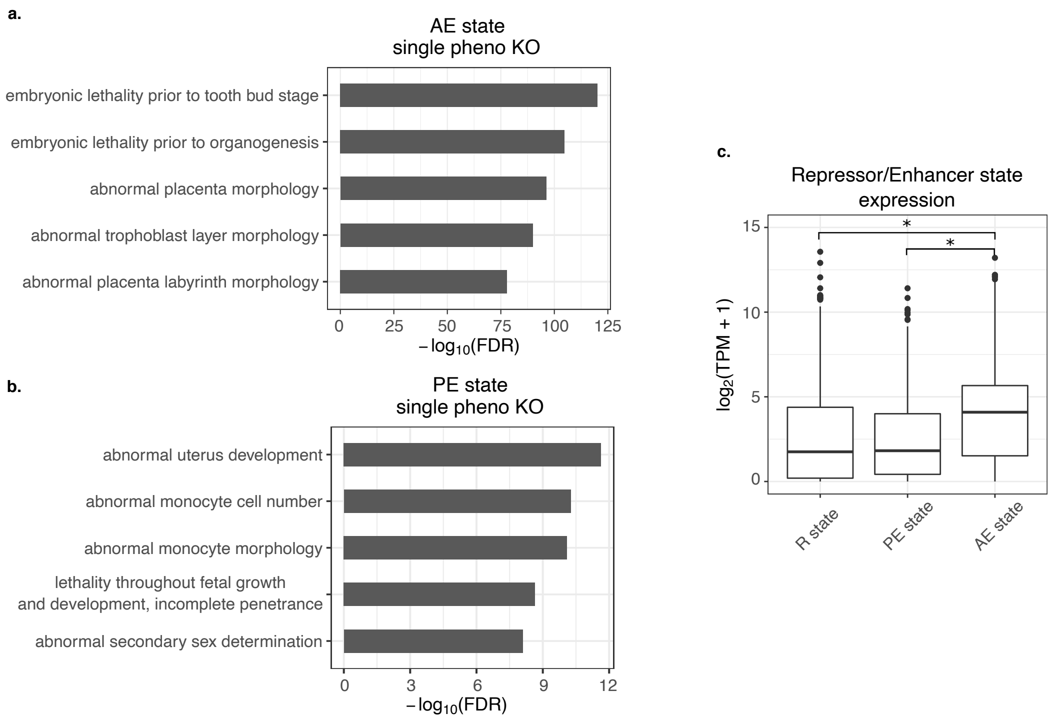

Supplementary Figure S3. (a) Genes associated with the AE state are enriched for terms related to placenta morphology and embryonic lethality when using the Mouse Phenotype Single KO Ontology. (b) Genes associated with the PE state are enriched for monocyte and early development terms when using the Mouse Phenotype Single KO Ontology. (c) Boxplot showing significantly higher expression of genes associated with the AE state than genes associated with the R or PE states using e9.5 placenta RNA-seq data[1] (* p-value < 0.05; students one-tailed t-test).

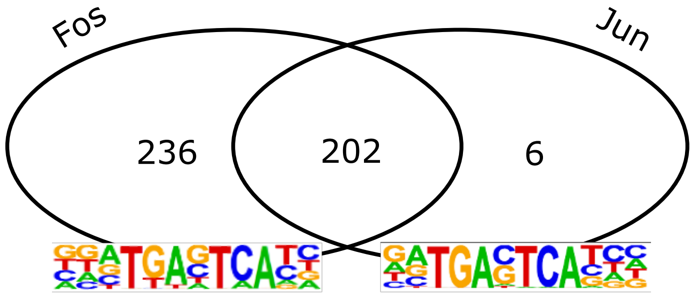

Supplementary Figure S4. Venn-diagram showing the number of regions associated the FOS motif (left), the JUN motif (right) or both.

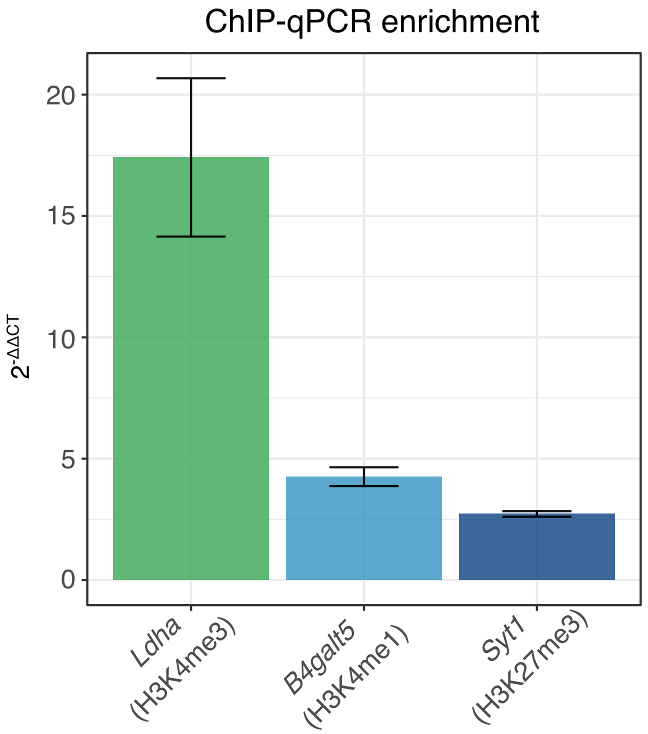

Supplementary Figure S5. ChIP-qPCR enrichments, where enrichment was calculated using the ΔΔCt method with 28S as a reference primer and input DNA as a control. Bar plot shows mean enrichment of the positive controls for each histone modification ChIP experiment.

1. Tuteja, G., Chung, T. & Bejerano, G. Changes in the enhancer landscape during early placental development uncover a trophoblast invasion gene-enhancer network. *Placenta* **37**, 45–55 (2016).
2. Hanna, C. W. *et al.* Endogenous retroviral insertions drive non-canonical imprinting in extra-embryonic tissues. *Genome Biol.* **20**, (2019).
3. Starks, R. R., Biswas, A., Jain, A. & Tuteja, G. Combined analysis of dissimilar promoter accessibility and gene expression profiles identifies tissue-specific genes and actively repressed networks. *Epigenetics Chromatin* **12**, 16 (2019).
